# The future of health markets

**DOI:** 10.1186/1744-8603-10-51

**Published:** 2014-06-24

**Authors:** Sara Bennett, Gerald Bloom, Jeffrey Knezovich, David H Peters

**Affiliations:** 1 Johns Hopkins School of Public Health, 615 North Wolfe St, Baltimore, MD, USA; 2 Institute of Development Studies, University of Sussex, Brighton, UK

## 

This editorial introduces a new open collection in Globalization and Health on the topic of the future of health markets. In 2012, we undertook work for the Rockefeller Foundation that sought to inform their future investments in health markets. Whereas health markets are sometimes viewed solely through the lens of private health care providers, our approach was broader, viewing such providers as one of many inter-connected actors who work within a market system [1]. We were interested in better understanding the interactions between these many actors, including public and private health care providers, payors, regulators, and civil society organizations, and the rules and regulations that govern the market. Our starting point was that poor people in low- and middle-income countries (LMICs) frequently depend on care from private health care providers, particularly informal private providers, and so, to expand access to high quality services for the poor, we need to think more strategically about how to influence health markets [2,3].

Health markets are the subject of increasing attention, and there are a number of factors driving this. One such driver is economic growth in some LMICs, which has led to rapid market growth [4]. Other factors include changes in views on the role of the state and markets and the emergence of diverse models of organizing service provision, particularly in emerging economies such as India [5]. The current focus on universal health coverage and the associated expansion of health insurance may also have significant impacts on health markets [6]. In particular, there appears to be increasing consensus that if government can finance quality services for the poor, then it matters less who provides those services. Further the growth of pooled funding for health provides significant opportunities for monitoring and improving quality of care in health markets. Finally technological change is also driving market developments [7]. For example, increased access to and more sophisticated applications of information and communication technologies (ICTs) along with low-cost diagnostic technologies have facilitated the emergence of new kinds of health provider networks, some bridging formal and informal providers. Further, ICTs may stimulate more “direct to client” marketing than we have seen previously.

As part of the work conducted for the Rockefeller Foundation, we surveyed a number of global stakeholders, including representatives of international organizations and foundations, private sector firms, non-governmental organizations and academics to better understand what they perceived to be the critical frontiers for health markets in the future: where were the pressure points in health markets that could trigger positive change across the market? Three different themes emerged from this survey:

***• The role of private for-profit providers*** – there was a strong sense from stakeholders that formal sector, private for-profit providers were unlikely to serve the poor if left on their own, and that the only way in which to get such providers to reach this segment of the market was through subsidies, from government or other sources, or through the expansion of health insurance schemes. Nonetheless there was thought to be a rapid evolution in the nature of business models that private providers were pursuing and a desire to better understand the implications of these emerging business models.

***• Regulation*** - Health markets in LMICs continue to be plagued by poor quality and unnecessary care, and non-transparent pricing. Regulatory failure, including inappropriate regulations, excessive regulations and weakly enforced regulations, were viewed to be a core part of this problem, and many respondents saw strengthening of appropriate regulatory structures to be a critical challenge facing health markets in the years to come. Unfortunately there appear to be relatively few successful innovations in regulation from which to learn lessons, highlighting an important need for future research and policy intervention.

***• Learning*** - The evidence base on many health market issues is relatively weak, and the differences across countries in market structure mean that it is difficult to transfer findings from one setting to another. Many respondents were frustrated by the lack of evidence on which to base strong policy recommendations. However respondents felt that new types of research designs and approaches were needed and simply investing in “more of the same” type of research would not solve problems.

Throughout all of these themes runs the notion that strong health markets require strong government capacity to develop and implement appropriate policy frameworks and engage with the private sector. Many of these issues were discussed during a meeting held in Bellagio in December 2012 that addressed the Future of Health Markets, a brief summary of which is available online [8].

The papers included in this collection reflect these three themes. Tung and Bennett [9] address the relevance of the Bottom of the Pyramid model to health, and conclude that without support from government, or the expansion of health insurance schemes, large private for-profit providers in the health sector are unlikely to address the needs of the poor. Bloom et al. [10] review the scope for innovations in health regulation, and what needs to be done to strengthen health market regulation in an appropriate fashion. Bennett et al. [11] consider learning about health markets, the sources of data available, and the use of evidence in policy and practice, and describe steps that could be taken to advance this. Finally the round table discussion [12] brings together perspectives from a diversity of stakeholders, many of whom participated in the Bellagio meeting, on what the key challenges in health markets are in the future.

This will be an open collection: authors are welcome to submit additional papers on the future of health markets. Papers that build on the themes and issues discussed here in the editorial and accompanying papers would be particularly appreciated, as would papers that address different perspectives on the future of health markets – perhaps documenting trends in health markets, exploring the nature of new provider networks that are emerging, or ICT innovations to link formal and informal sector providers. All papers intended for this special collection should signal this in their covering letter, and will go through Globalization and Health’s usual review process. We look forward to stimulating a lively discussion about future health markets.

## Competing interests

This work was funded by the Rockefeller Foundation and the UK Government through UK aid. The authors declare no further competing interests.

## Authors’ contributions

SB, GB, DP jointly conceived the work described here. SB drafted the first version of the manuscript. All authors reviewed, critically revised and approved the final manuscript.
